# Effect of rifampicin combination-regimens against multi-drug resistant strains in North India

**DOI:** 10.6026/97320630018482

**Published:** 2022-05-31

**Authors:** Misbahuddin M Rafeeq, Alaa Hamed Habib, Ahmad Alzamami, Norah A Alturki, Mutaib M Mashraqi, Youssef Saeed Alghamdi, Saleh Alshamrani, Afaf Awwadh Alharthi, Suhail Ahmad

**Affiliations:** 1 Department of Pharmacology, Faculty of Medicine, Rabigh, King Abdulaziz University, Jeddah, 21589, Saudi Arabia; 2 Department of Physiology, Faculty of Medicine, King Abdulaziz University, Jeddah, 21589, Saudi Arabia; 3 Clinical Laboratory Science Department, College of Applied Medical Science, Shaqra University, AlQuwayiyah 11961, Saudi Arabia; 4 Clinical Laboratory Science Department, College of Applied Medical Science, King Saud University, Riyadh 11433, Saudi Arabia; 5 Department of Clinical Laboratory Sciences, College of Applied Medical Sciences, Najran University, Najran 61441, Saudi Arabia; 6 Department of Biology, Turabah University College, Taif University, P.O.BOX 11099, Taif 21944, Saudi Arabia; 7 College of Applied Medical Sciences, Department of Clinical Laboratory Sciences, Taif University, Taif, Saudi Arabia; 8 Department of Bioengineering, Integral University, Lucknow, India

**Keywords:** Antibiotic combinations, Cefotaxime, DNA fragmentation assay, Escherichia coli

## Abstract

It is well-acknowledged that 'combination therapy' of antibiotics is indispensable for the treatment of patients suffering from serious bacterial infections. Therefore, it is of interest to collect data from the in vitro tests using 'rifampicin-cefotaxime' and 'rifampicin-tetracycline' combination regimens against multi drug resistant Escherichia coli and Klebsiella pneumoniae strains of nosocomial source in order to determine the effectiveness of the combination therapy. The minimum inhibitory concentration (MIC) values for cefotaxime, tetracycline and rifampicin antibiotics were found to be comparatively high for each of the antibiotics when given individually. However, carefully prepared combination-regimens exhibited significant inhibitory effect on the same bacterial isolates. DNA fragmentation study confirmed that 'rifampicin-cefotaxime' and 'rifampicin-tetracycline' combination-regimens could cause breakage of the bacterial DNA. Thus, we show that combination-regimens namely, 'rifampicin-cefotaxime' and 'rifampicin-tetracycline' were found to be capable of maintaining rifampicin susceptibility in the E. coli and K. pneumoniae strains. However, this susceptibility was not maintained by only rifampicin. More data using animal model experiments are needed for confirming and deriving translational benefits from these findings in future.

## Background:

Infectious diseases occurring due to multidrug resistant Gram-negative bacteria are a major therapeutic challenge [1, 2] in community as well as hospital settings [3]. If we consider the population living in the developed nations, the average life tenure for an individual has increased. Albeit this augmented tenure is also coupled to many other health issues because of steep upsurge in the cases of obesity and diabetes. Accordingly, this has resulted in the rise of 'chronic wound-infections'. In USA, such infections had been estimated to touch some 6.5 x 106 individuals and it was projected to produce a burden of 25 x 109 US$ per annum, along with a high chance of further increase in the years to come [4]. In order to overcome/decrease the pace of drug-resistance, disease treatment employing 'combination-regimens' of carefully selected antibiotics is a routine practice in many health care centers [5-7]. This type of approach could lead to a sort of synergism thereby resulting in overall enhanced efficiency of the treatment as well as decrease in the total amount of each drug consumed. Moreover, the chance of many contra-indications could also be minimized. Most importantly, this could duly decrease the total expenditure incurred [8, 9]. Furthermore, it had been argued that using combination-regimens i.e. including more than one antibiotic, each having a different 'action-mode' and/or 'target' might reduce the chance of emerging drug-resistance while the treatment continued [10]. As far as the case of chronic infections is considered, it is of particular importance, as the drug-therapy associated with such treatment may be significantly prolonged. A couple of substitute approaches to increase the effectiveness of known antibiotic treatments against hardier bacteria had been also suggested. For instance, augmenting the drug-toxicity in a synergistic style by combining these antibiotics to other chemicals or to metals possessing dissimilar targets is quite a germane approach. This line of attack on bacteria expressively decreases the chance of appearance of bacteria which could concurrently resist both of the anti-bacterial [11]. Therefore, it is of interest to document data from the in vitro tests using 'rifampicin-cefotaxime' and 'rifampicin-tetracycline' combination regimens against multi drug resistant Escherichia coli and Klebsiella pneumoniae strains of nosocomial source in order to determine the effectiveness of the combination therapy.

## Materials and Methods:

### Bacterial isolates:

The bacterial strains (E. coli and K. pneumoniae) were obtained from cultured urines on selective media. We used the reference methods described by Cowan and Steel [12] to isolate and identify the bacterial strains.

### Broth-dilution test:

We used an adapted protocol that was based on the famous macro broth dilution protocol as described by Ibrahim et al. [13] to establish the values for minimum inhibitory-concentration (MIC). Bacterial culture which was incubated for 12 hours in nutrient broth was diluted 100 folds using sterile nutrient broth (100µl of bacterial-cultures added to 10ml of nutrient broth that had 105cfu of bacteria). Progressively, we added increasing volumes of different concentration of antibiotics and their combinations to the test-tubes having the bacterial-cultures to measure the inhibiting-concentration in a particular tube inhibiting the bacterial-growth. These test-tubes were incubated at 37°C for 18-24 hours. We inspected these test-tubes for visible turbidity while the O.D. values were measured at a wavelength of 620nm. Sterile nutrient broth was used as a control. The minimum concentration which inhibited the visible-growth of the bacterial strains was noted as the corresponding MIC-value. Further, MICs for the selected antimicrobials were tested by HiComb MIC test strips (Hi-media, India).

### Agar well-diffusion test:

Agar well-diffusion test was used to assess the antibacterial effects of the antibiotics and their combinations [14]. Nutrient-agar was duly added to all of the Petri-plates which had a diameter of 9 cm. After the solidification of the agar, pertinent number of wells, each having a 5 mm diameter, were prepared in the agar plate(s). 100µl (105cfu) of each of the diluted bacterial culture was inoculated onto the nutrient agar-plates with the aid of sterile cotton-swabs. Rifampicin, tetracycline, cefotaxime as well as their combinations (rifampicin-cefotaxime and rifampicin-tetracycline) were added to each of the bored wells in the agar plates. These were allowed to diffuse at normal room temperature for about 20 minutes. Post-incubation at 37°C for 24h, all of the plates were scrutinized for 'growth inhibition-zones' along with duly measuring the respective zone-diameters.

### DNA fragmentation test:

DNA fragmentation assay was carried out under stress conditions at different concentrations of cefotaxime, tetracycline and rifampicin and their combinations (rifampicin-cefotaxime and rifampicin-tetracycline) employing the method of Fernandez et al. [15].

## Results and Discussion:

### Minimum inhibitory concentration (MIC) determination:

Antibacterial activity of cefotaxime, tetracycline and rifampicinand their combinations (rifampicin-cefotaxime and rifampicin-tetracycline) were evaluated against Gram-negative (E. coli and K. pneumoniae) bacterial strains. MIC measurement was performed employing an adapted version of the famous macro-broth dilution protocol [13]. MIC values of cefotaxime, tetracycline and rifampicin were found to be individually high for E. coli and K. pneumoniae strains. The MIC-values of cefotaxime, tetracycline and rifampicin for E. coli (E1) were found to 15.625, 31.25, and 7.81 mg/L, respectively, whereas the same for K. pneumoniae (K1) were determined to be 31.25, 7.81, 15.62 mg/L, respectively. In contrast to this, the MIC value of rifampicin combined with cefotaxime or tetracycline was found to be comparatively low for E. coli and K. pneumoniae strains (Table 1 - see PDF). MIC-values corresponding to 'rifampicin-cefotaxime' and 'rifampicin-tetracycline' combinations for E. coli (E1) were found to be 0.97 and 1.95 mg/L, respectively, whereas the same for K. pneumoniae (K1) were found to be 0.97 and 1.95 mg/L, respectively. Our results are consistent with the findings in another study where the author has described results of combination therapy of rifampicin with other antibiotics against multi drug resistant Pseudomonas aeruginosa isolates [16]. Furthermore, a combination of rifampicin with penicillin or ampicillin was established to exhibit a full synergistic bactericidal activity way back in 1982 by Tuazon et al. [17]. Further, the MICs of the different antibiotics were determined on both E. coli and K. pneumoniae isolates as presented in table 2 (Figure 1 - see PDF). Very high MICs were obtained for amoxycillin, ceftazidime, cefepime, erythromycin and ceftriaxone indicating that the studied strains were highly resistant to these antibiotics. The MICs of aztreonam, amikacin, ciprofloxine and streptomycin were moderate, but still in the resistant range. The MIC values obtained against different antibiotics in our study were in due agreement with earlier reports [18-20].

### Determination of antimicrobial effect:

The 'Zone of Inhibition'-tests revealed that all of the bacterial isolates (nosocomial strains of E. coli and K. pneumoniae) were not susceptible to the solo drugs i.e. rifampicin, cefotaxime and tetracycline, when given individually (Table 3 - see PDF). However, the antibiotic combination regimens exhibited significant inhibitory effect on E. coli as well as K. pneumoniae isolates. The zones of inhibition corresponding to 'rifampicin-cefotaxime' and 'rifampicin-tetracycline' combinations against E. coli (E1) were found to possess diameters of 20 and 17 mm, respectively; while the same for K. pneumoniae (K2) were found to have diameters of 19 and 17 mm, respectively. Therefore, it can be concluded that 'rifampicin-cefotaxime' and 'rifampicin-tetracycline' combinations displayed potent antimicrobial activity against the tested isolates. The zones of inhibition shown by cefotaxime, tetracycline, rifampicin and their combinations (rifampicin-cefotaxime and cefotaxime-tetracycline) on E. coli and K. pneumoniae strains are shown in Figure 2 and Figure 3(see PDF). In a notable study which supports the findings described herein, the authors have reported that the combination of rifampicin with other selected antibiotics demonstrated full synergistic bactericidal activity [17].

Rifampicin has a number of characteristics that might make it significantly effective when used in combination with other antibiotics, namely, its potent bactericidal activity [21], modest activity against non-growing cells [22], ability to penetrate the cells [23] and a variety of tissues and compartments, such as cerebrospinal fluid and bone [24]. Hence, our data supports the idea of designing 'rifampicin-cefotaxime' as well as 'rifampicin-tetracycline' combination regimens for the effective treatment of multidrug-resistant strains of E. coli and K. pneumoniae of clinical origin.

### DNA fragmentation assay:

DNA fragmentation study was carried out using rifampicin, cefotaxime, tetracycline and their combinations ('rifampicin-cefotaxime' and 'rifampicin-tetracycline') on DNA of the aforementioned bacterial isolates. We observed that rifampicin-cefotaxime and rifampicin-tetracycline combinations are lethal for bacterial DNA as shown in Figure 4 and Figure 5(see PDF). In fact, rifampicin acts by inhibiting the DNA-dependent-RNA-polymerase, thereby preventing expression of the bacterial genes [25]. However, in the current study some extent of DNA fragmentation was also observed in cefotaxime and tetracycline treated isolates. Accordingly, Molina-Quiroz et al. [26] studied synergistic effect of tellurite/cefotaxime in E. coli. They showed that the tellurite/cefotaxime treatment caused cellular damage. Increased levels of intracellular superoxide and OH produced by tellurite and cefotaxime, respectively, generated direct damage to DNA [26]. In contrast to this, tetracycline has been shown to induce SOS response in Vibrio cholera [27]. The SOS response is an inducible pathway governing DNA repair that was first described in E. coli [28].

Fragmentation of the Chromosomal-DNA might directly or indirectly relate to the phenomenon of death of the cell. As opposed to DNA-fragmentation concerning higher eukaryotic-cells, fragmentation of the same in microbes has been explored to somewhat lesser extent. In fact, Chromosomal fragmentation of DNA of chromosomal origin might represent cell-death because this in turn is the outcome of substantial breaks in the double strand of the DNA. In higher-cells, the said observation might be a sequel to an active 'programmed-cell-death/apoptosis', where the DNA is cleaved via an activated endonuclease [29]. Else, this fragmentation could also occur passively by necrotic cell-death. However, the passive-type fragmentation of the DNA occurs more frequently in the microbes that get destroyed due to varied reasons. Nevertheless, researches indicate a feasibility of occurrence of 'programmed cell death/apoptosis' in single-celled bacterial pathogens as well [30]. In actual fact, bactericidal-drugs might activate an apoptosis like pathway. Scientists have noted that bactericidal-drugs could trigger the formation of hydroxyl-radicals which in turn could underpin cell death. It has been argued that tolerant-type bacteria, that are somewhat resistant to certain bactericidal-drugs, might actually be the bacteria that possess a disabled apoptotic pathway/program [31].

## Conclusion:

Combination-regimens of 'rifampicin-cefotaxime' and 'rifampicin-tetracycline' were found to be capable of maintaining rifampicin susceptibility in the E. coli and K. pneumoniae strains unlike solo rifampicin. More data from animal model experiments are needed for confirming and deriving translational benefits from these findings in future.
